# Effectiveness of Virtual Reality on Balance and Risk of Falls in People with Multiple Sclerosis: A Systematic Review and Meta-Analysis

**DOI:** 10.3390/ijerph192114192

**Published:** 2022-10-30

**Authors:** Ana Castellano-Aguilera, Gemma Biviá-Roig, Ferran Cuenca-Martínez, Luis Suso-Martí, Joaquín Calatayud, María Blanco-Díaz, José Casaña

**Affiliations:** 1 Exercise Intervention for Health Research Group (EXINH-RG), Department of Physiotherapy, University of Valencia, 46010 Valencia, Spain; 2 Department of Nursing and Physiotherapy, Faculty of Health Sciences, University CEU-Cardenal Herrera, CEU Universities, 46115 Valencia, Spain; 3 Surgery and Medical Surgical Specialities Department, Faculty of Medicine and Health Sciences, University of Oviedo, 33003 Oviedo, Spain

**Keywords:** virtual reality, multiple sclerosis, neurorehabilitation, balance, risk of falls

## Abstract

The aim of this study was to systematically review the scientific evidence related to the physiotherapy interventions in neurorehabilitation that utilize virtual reality (VR) for balance training and risk of falls in people with multiple sclerosis (MS). A search was conducted in Medline (PubMed), PEDro, and Google Scholar to identify all the relevant studies. Clinical trials assessing the effects of VR in people with MS were included. Risk of bias was evaluated using the Cochrane Risk of Bias Tool and PEDro scale. Qualitative analysis was performed according to the GRADE. In total, 16 studies (n = 663) were included. The meta-analysis showed statistically significant differences for the VR intervention in comparison with conventional treatment for balance, with a moderate clinical effect in eight studies (SMD: 0.63; 95% CI 0.34–0.92; *p* < 0.05). In addition, the meta-analysis showed statistically significant differences for the VR intervention in comparison with conventional treatment for risk of falls, with a small clinical effect in six studies (SMD: −0.55; 95% CI −1.07–0.04; *p* < 0.05). VR-based treatments are more effective than non-intervention in improving balance and fall risk in people with MS, with a very low certainty of evidence. In addition, they also show to be more effective than conventional rehabilitation, with a very low certainty of evidence.

## 1. Introduction

Multiple sclerosis (MS) is an autoimmune, chronic, progressive, and neurodegenerative disease of the central nervous system [1]. These processes produce a loss of myelin in the white matter of the cerebral hemispheres, cerebellum, brainstem, spinal cord, and optic nerves that lead to behavioral, cognitive, sensory, and motor symptoms [2]. The main symptoms of MS include the decrease of the capability to hold one’s balance and coordination. Specifically, 89% of MS patients usually show motor symptoms, such as muscle weakness, balance, coordination, or gait problems, while 87% show sensory symptoms, such as visual disturbances or pain (burning, electrical, and sharp sensations). In addition, 83% of MS patients show fatigue problems, 40–70% of MS patients show cognitive impairment, and 30–45% of patients show depressive symptoms [3]. At the same time, a directly proportional relationship between the balance deficit and the fall increase in MS patients has been demonstrated [4]. Risk of falls is considered one as the most disabling symptoms, given that it reduces one’s mobility and independence, so it directly influences one’s quality of life, decreasing it notably [5,6].

There is no definitive cure for MS, but there are multiple therapies focused on improving the functionality of the patients after relapse and preventing a greater future disability. Nonetheless, therapies that include both medication and neurorehabilitation can improve symptoms, but neither prevent the onset of the pathology, nor end its destruction [7,8]. Conventional physiotherapy for patients with neurological impairment often includes physical exercises related to motor skill practice, which are repetitive and may not motivate some patients. Lack of motivation may decrease adherence to treatment [9]. Given these drawbacks, in the last years there has been a boom in physiotherapy in neurorehabilitation based on virtual reality (VR). This method allows for an individualized training that is high intensity, multisensorial, and task orientated, and at the same time, it provides instantaneous feedback to the patient [10,11,12,13]. Likewise, it has been demonstrated that VR helps to improve the neuropsychological deficit by stimulating and boosting the cerebral plasticity in the neurological population [14]. For example, VR has been proposed as an adequate physiotherapy treatment for motor and cognitive rehabilitation in stroke patients, given that this method motivates the patients to continue practicing the exercises and can contribute to a greater motor learning and visual, auditive, and tactile practice [15].

Regarding the role of VR in MS, different systematic reviews provide insights into the current state-of-the-art methods. For example, Casuso-Holgado et al. [16] studied three outcome measures: postural control in three conditions (bipedal eyes opened tests, bipedal eyes closed tests, and unipedal eyes opened tests), functional balance, and walking speed. The main results showed that in postural control, VR was significantly superior to no intervention in the improvement of bipedal eyes opened tests, but not if VR was compared against conventional training. VR was not also significantly superior to conventional training in the improvement of bipedal eyes closed tests nor in unipedal eyes opened tests. In addition, VR was not statistically superior to no intervention or to a standard balance intervention in the improvement of functional balance or walking speed. In addition, Grazia-Maggio et al. [17] and Massetti et al. [18] published systematic reviews without meta-analysis and showed that MS patients presented a clinical improvement in motor, such as gait and balance outcome measures, and cognitive function. However, Moreno-Verdú et al. [19] showed that VR was as effective as conventional training for improving balance in patients with MS, but no data suggested that VR was superior to other interventions in improving gait speed. Similarly, Cortés-Pérez et al. [20] showed that VR improves relevant variables in MS as fatigue and quality of life, and Akkan et al. [21] found that VR could reduce fear of falling in MS patients.

Thus, analyzing the slightly mixed results between these reviews [16,17,18,19], together with the fact that since the last meta-analysis [16], about 30% more controlled trials have been reported evaluating the role of VR in people with MS, we believe that more research is needed to clarify the role of VR in improving clinical variables in MS. The main aim of this article was to systematically review and summarize the scientific evidence related to physiotherapy interventions that utilize VR for the balance training and risk of falls in people with MS.

## 2. Materials and Methods

This systematic review and meta-analysis is reported according to the Preferred Reporting Items for Systematic Reviews and Meta-analyses guidelines described by Moher et al. [22].

### 2.1. Search Strategy

The search for studies was performed using Medline (PubMed), PEDro, and Google Scholar. The final search was run on 15 September 2020. The search equations can be found in Supplementary Materials S1.

We employed a validated search filter and adapted it to all of the databases [23,24,25]. Based on international criteria, we applied no restriction with respect to the language of the studies [26]. Reviewers were fluent in English and Spanish, and a professional interpreter was used where necessary. Using the same methodology, two reviewers conducted the search for the studies independently (ACA and JCG). Consensus was reached through the participation of a third-party reviewer (LSM). We employed a manual search through journals that typically publish on the topic in question to include all available studies. For all the studies found in the first search, we reviewed the introduction, discussion, and reference sections so as not to overlook any relevant studies.

### 2.2. Inclusion Criteria

The selection criteria employed in this systematic review and meta-analysis were based on methodological and clinical factors, such as the Population, Intervention, Control, Outcomes, and Study Design (PICOS) variable described by Stone et al. [27] Studies were considered eligible for this review if they were randomized controlled trials; included participants from both sex and older than 18 years with the diagnostic of MS; investigated the efficacy of VR as an independent intervention or in combination with other interventions compared to usual care or standard rehabilitation (i.e., physical therapy, exercise intervention) combined or with placebo interventions; and considered outcome measures related to static and dynamic balance as well as risk of falls.

Two independent reviewers (LSM and FCM) used a set of predetermined criteria to independently examine the titles, abstracts, and keywords of studies generated by the searches. We retrieved full-text publications for all abstracts of potential interest. Two review authors then independently examined the full-text reports to determine whether the studies met the selection criteria. Consensus was reached through the participation of a third-party reviewer (JCG) [28]. Data described in the results were extracted by means of a structured protocol that ensured that the most relevant information was obtained from each study [29].

### 2.3. Methodological Quality Assessment

#### 2.3.1. Risk of Bias

Two authors independently evaluated the risk of bias of each included study, using the Cochrane “Risk of bias” tool (version 5.1.0) (OR also known as seven-criteria Cochrane risk of bias tool).

This tool has seven domains: selection bias (random sequence generation, allocation concealment), performance bias (blinding of participants and personnel), detection bias (blinding of outcome assessment), attrition bias (incomplete outcome data), reporting bias (selective reporting), and other potential sources of bias. Each domain was scored as “yes”, “no”, or “unclear” and classified into one of three categories as “high risk of bias”, “low risk of bias”, or “unclear”.

Two independent reviewers (LSM and FCM) examined the quality of the selected studies using the same methodology. Disagreements between the reviewers were resolved by consensus with a third reviewer (ACA). The concordance between the results (inter-rater reliability) was performed using Cohen’s kappa coefficient (κ): κ > 0.7 indicates a high level of agreement between assessors, κ = 0.5–0.7 indicates a moderate level of agreement, and κ < 0.5 indicates a low level of agreement [30].

#### 2.3.2. PEDro Scale Assessment

The studies’ methodological quality was assessed using the PEDro scale [31], which assesses the internal and external validity of a study and consists of 11 criteria: (1) specified study eligibility criteria, (2) random allocation of participants, (3) concealed allocation, (4) measure of similarity between groups at baseline, (5) patient blinding, (6) therapist blinding, (7) assessor blinding, (8) fewer than 15% dropouts, (9) intention-to-treat analysis, (10) intergroup statistical comparisons, and (11) point measures and variability data.

The methodological criteria were scored as follows: yes (1 point), no (0 points), or do not know (0 points). The PEDro score for each selected study provided an indicator of the methodological quality (9–10 = excellent; 6–8 = good; 4–5 = fair; 3–0 = poor) [32].

### 2.4. Certainty of Evidence

The certainty of evidence analysis was based on classifying the results into levels of evidence according to the Grading of Recommendations, Assessment, Development, and Evaluation (GRADE) framework, which is based on five domains: study design, imprecision, indirectness, inconsistency, and publication bias [33]. The assessment of the five domains was conducted according to GRADE criteria and performed by two independent reviewers [34,35]. Evidence was categorized into the following four levels accordingly. (a) High quality: Further research is very unlikely to change our confidence in the estimate of effect. All five domains are also met. (b) Moderate quality: Further research is likely to have an important impact on our confidence in the estimate of effect and might change the estimate of effect. One of the five domains is not met. (c) Low quality: Further research is very likely to have an important impact on our confidence in the estimate of effect and is likely to change the estimate. Two of the five domains are not met. (d) Very low quality: Any estimate of the effect is very uncertain. Three of the five domains are not met [34,35]. The recommendations could be increased or decreased in each domain according to the following criteria. Regarding the study design domain, the recommendations were downgraded one level in case there was an uncertain or high risk of bias and serious limitations in the estimate of the effect (more that 25% of the participants were from studies with low methodological quality). Regarding inconsistency, recommendations were downgraded one level when the I^2^ was substantial or large (>50%). For indirectness evidence, domain recommendations were downgraded when severe differences in interventions, study populations, or outcomes were found (recommendations were downgraded in absence of direct comparisons between the interventions of interest or when there are no key outcomes, and the recommendation is based only on intermediate outcomes or if more than 50% of the participants were outside the target group). In relation to imprecision, domain recommendations were downgraded one level if there were n < 300 participants for continuous data.

### 2.5. Data Synthesis and Analysis

We employed the inverse variance method and random effects model for all the studied variables [36]. We evaluated the statistical heterogeneity using the Chi-squared test (with statistical significance set at *p* < 0.10) and measured heterogeneity by calculating the inconsistency index (I^2^) [37]. An I^2^ between 0% and 40% might not be important heterogeneity, an I^2^ between 30% and 60% may represent moderate heterogeneity, and an I^2^ between 50% and 90% is considered to represent substantial heterogeneity. Finally, I^2^ = 75–100% would involve considerable heterogeneity [37]. We calculated the effect sizes by the standardized mean difference (SMD) for all variables given that they are expressed in different scales and units and set the confidence intervals at (95% CI). The estimated SMDs were interpreted as described by Hopkins et al. [38] (i.e., an SMD of 4.0 was considered to represent an extremely large clinical effect; 2.0–4.0 represents a very large effect; 1.2–2.0 represents a large effect; 0.6–1.2 represents a moderate effect; 0.2–0.6 represents a small effect; and 0.0–0.2 represents a trivial effect).

To detect publication bias and to test the influence of each individual study, we performed a visual evaluation of the funnel plot, seeking asymmetry. We employed MetaXL software for the quantitative analysis (version 5.3 (EpiGear International, Sunrise Beach, Queensland, Australia)), using the same three inclusion criteria for the systematic review and meta-analysis: the results showed detailed information regarding the comparative statistical data of the exposure factors, therapeutic interventions, and treatment responses; the intervention was compared with a similar control group (e.g., usual care or standard rehabilitation); and data on the analyzed variables were represented in at least three studies.

## 3. Results

**Figure 1:**
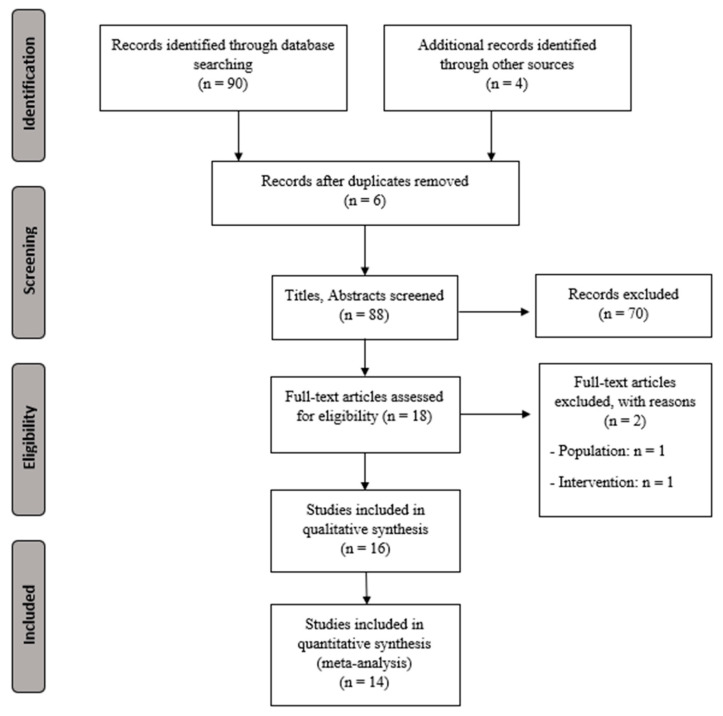
PRISMA flowchart for selecting studies.

Figure 1 shows the study flow chart and Table 1 presents the characteristics for the extracted data (sample size, demographic characteristics, intervention, outcomes, main results, and conclusions).

### 3.1. Study Characteristics

In the total included studies, a total of 663 participants with MS were included. The vast proportion of the studies compared VR-based interventions with traditional balance exercise rehabilitation over a period of 6 to 12 weeks. Five studies used VR using the Wii-Fit System, two used Microsoft Kinect, and the rest used proprietary systems.

### 3.2. Methodological Quality Analysis

**Figure 2:**
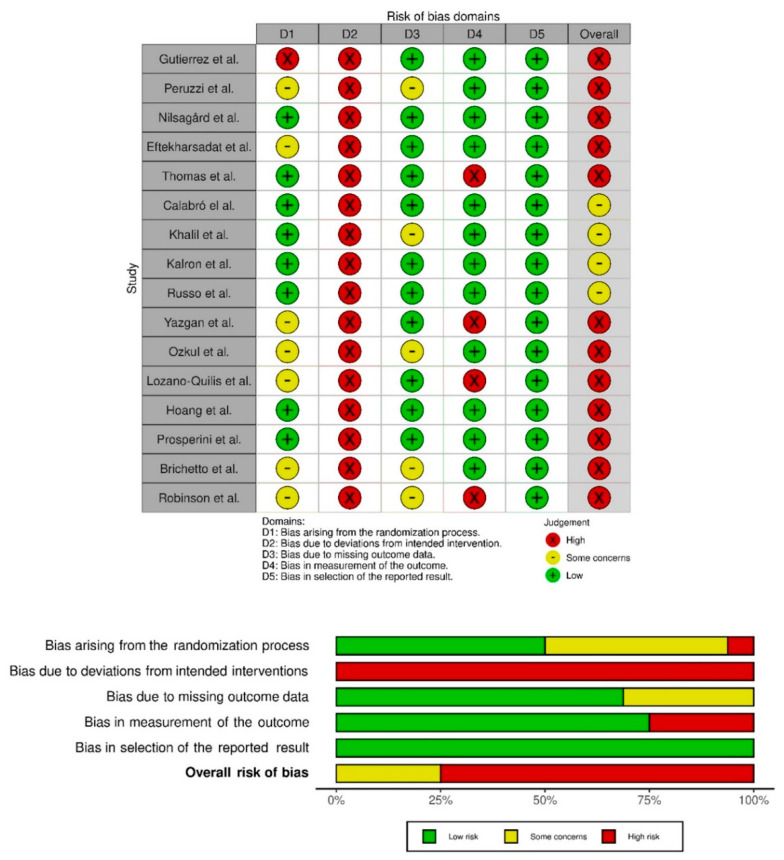
Risk of bias summary. Review authors’ judgements about each risk of bias item for each included study (Risk of Bias scale) and risk of bias graph. Review authors’ judgements about each risk of bias item presented as percentages across all included studies (Risk of Bias scale) [9,39,40,41,42,43,44,45,46,47,48,49,50,51,52,53].

The studies’ quality was evaluated with the Cochrane assessment tool. Most of the studies had a low risk of selective reporting bias. The domain with the highest percentage of studies with a high risk of bias was the blinding of participants and personnel (performance bias). Figure 2 show the summary of the risk of bias and the graph for the risk of bias, respectively. The inter-rater reliability of the methodological quality assessment between assessors was high (κ = 0.81). Table 2 lists the PEDro scores for each study.

### 3.3. Meta-Analysis Results

#### 3.3.1. Balance (BBS)

**Figure 3:**
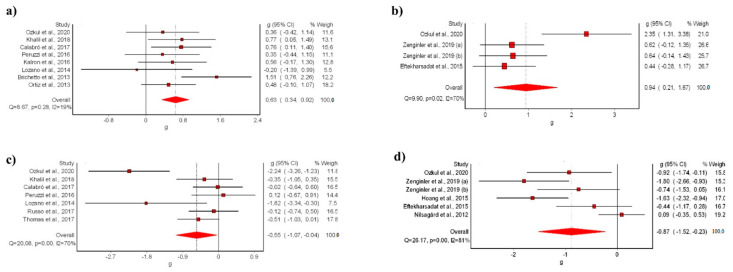
Synthesis forest plot of: (**a**) BSS variable (VR vs. conventional treatment); (**b**) BSS variable (VR vs. no-treatment); (**c**) TUG variable (VR vs. conventional treatment); (**d**) TUG variable (VR vs. no-treatment). The forest plot summarizes the results of the included studies (sample size, standardized mean differences (SMDs), and weight). The small boxes with the squares represent the point estimate of the effect size and sample size. The lines on either side of the box represent a 95% confidence interval (CI) [9,39,40,41,42,43,44,45,46,47,48,49,50,51,52,53].

The meta-analysis showed statistically significant differences for the VR intervention in comparison with conventional treatment, with a moderate clinical effect in eight studies [9,39,40,42,44,45,47,48] (SMD: 0.63; 95% CI 0.34–0.92; *p* < 0.05) without evidence of significant heterogeneity (Q = 8.67, *p* < 0.05, I^2^ = 19%) (Figure 3a). The shape of the funnel and DOI plot did not present asymmetry, and the LFK index showed no asymmetry (LFK, −0.4) indicating a low risk of publication bias (Supplementary Materials S2). The certainty of evidence was low, showing that VR likely increases balance in people with MS in comparison to conventional rehabilitation, with evidence being downgraded due to imprecision (sample size < 300) and risk of bias (Table 3).

The meta-analysis showed statistically significant differences for the VR intervention in comparison with no-treatment, with a moderate clinical effect in three studies [41,47,53] (SMD: 0.94; 95% CI 0.21–1.87; *p* < 0.05) but with evidence of significant heterogeneity (Q = 9.90, *p* < 0.05, I^2^ = 70%) (Figure 3b). The shape of the funnel and DOI plot did not present asymmetry, and the LFK index showed minor asymmetry (LFK, 1.82) indicating a low risk of publication bias (Supplementary Materials S3). The certainty of evidence was very-low quality, showing that VR increases balance in people with MS in comparison to no-intervention, with evidence being downgraded due to imprecision (sample size < 300), risk of bias, and inconsistency (Table 3).

#### 3.3.2. Risk of Falls (TUG)

The meta-analysis showed statistically significant differences for the VR intervention in comparison with conventional treatment, with a small clinical effect in seven studies [9,40,45,47,48,51,52] (SMD: −0.55; 95% CI −1.07–0.04; *p* < 0.05) but with evidence of significant heterogeneity (Q = 20.08, *p* < 0.05, I^2^ = 70%) (Figure 3c). The shape of the funnel and DOI plot did not present asymmetry, and the LFK index showed minor asymmetry (LFK, −1–78) indicating a low risk of publication bias (Supplementary Materials S4). The certainty of evidence was very low quality, showing that VR reduces risk of falls in people with MS in comparison to conventional treatment, with evidence being downgraded due to imprecision (sample size < 300), risk of bias, and inconsistency (Table 3).

In addition, the meta-analysis showed statistically significant differences for the VR intervention in comparison with no-treatment, with a moderate clinical effect in five studies [41,43,46,47] (SMD: −0.87; 95% CI −1.52–−0.23; *p* < 0.05) but with evidence of significant heterogeneity (Q = 26.17, *p* < 0.05, I^2^ = 81%) (Figure 3d). The shape of the funnel and DOI plot presented asymmetry, and the LFK index showed minor asymmetry (LFK, −2.53) indicating a high risk of publication bias (Supplementary Materials S5). The certainty of evidence was very low quality, showing that VR reduces risk of falls in people with MS in comparison to no-intervention, with evidence being downgraded due to imprecision (sample size < 300), risk of bias, and inconsistency (Table 3).

## 4. Discussion

The results of this systematic review and meta-analysis suggest that VR can be considered as a valid and effective treatment for balance rehabilitation in people with MS, given that it has shown to be more effective than conventional treatment for increase balance and decrease risk of falls, with low to very low certainty of evidence. Nonetheless, given the considerable variety of therapeutic protocols and intensities implemented, the results need to be interpreted carefully.

Our findings are similar to those reported by Moreno-Verdú et al. [19] and Casuso-Holgado et al. [16] defending that VR can be beneficial as a complementary treatment to rehabilitate balance in MS patients. Casuso-Holgado et al. [16] only showed that VR balance training was more effective than no intervention with a moderate clinical effect (SMD = −0.64; 95% CI −1.05–0.24). However, the authors did not show significant effects of VR intervention compared with conventional treatment for the gait rehabilitation variable, in contrast with our meta-analysis results. In addition, Moreno-Verdú et al. [19] performed a systematic review in which they could only conclude that VR is as effective as conventional training but could not determine whether VR is more effective or not. Furthermore, the authors did not perform a statistical aggregation by meta-analysis. Therefore, this is the first study to determine that VR may be more effective than conventional rehabilitation in light of the new findings of the newly included studies.

At the same time, the results obtained in this study are in concordance with previous studies carried out in Parkinson patients [54] and stroke patients [55,56,57], considering that VR can be beneficial to improve balance as well as the cognitive recovery and motor performance. In addition, Maggio et al. showed that VR could boost motivation and participation in patients with MS, improving cognitive function (executive and visual-spatial abilities, attention, and memory skills) [17]. VR has some specific characteristics that may explain the results obtained in the present review. VR offers the possibility to perform a task-oriented training with a specific goal to improve motor variables like balance or gait ability. In addition, VR provides a real, simultaneous, and multisensorial feedback during the motor training, which allows patients to improve their performance through the learning of new motor strategies [44,58]. From a neurophysiological point of view, VR has been shown to activate the mirror neuron system, performing a visuo-motor transformation phenomenon through the activation of parietal areas and generating an efferent copy of the motor action, even though it is virtual [40]. This efferent copy can be stored in the central nervous system and could be used as a model to execute it later in a real way, increasing motor performance [40]. In addition, VR provides patients with MS multisensory feedback that could induce a sensorimotor neuroplasticity in the sensorimotor cortex, which could be related to a functional motor recovery [59]. Furthermore, VR can increase patients’ motivation during rehabilitation because the exercises performed in the virtual environment could be more fun than traditional neurological rehabilitation, promoting more motivation and adherence [60].

An additional consideration is the characteristics of the VR used, as well as the best parameters to optimize its use from a clinical point of view. In view of the results and previous literature in this field, it is not possible to determine a single best way of use. The great heterogeneity in VR systems, sessions performed, or intervention times makes it impossible to draw solid conclusions. However, it seems that the use of an avatar and a more realistic scenarios could strengthen the neuroplastic changes within higher sensory and motor areas belonging to the mirror neuron system [59,61].

It is important to mention that the results obtained in this investigation suggest that VR systems are easy and safe to use in the clinical setting, given that no incidents related to musculoskeletal injuries or disease outbreaks were recorded in any of the studies. So, we can conclude that this method opposes no risk for the patients. Moreover, this method has been shown to increase the adherence of the patients to the treatment, as the drop-out ratio was minimal, and the patients showed an increase in their positive attitude, trust, and physical and psychological state and a decrease in the level of disability.

### Limitations

This study has a number of limitations to be considered. First, although we followed a systematic search strategy, the risk of selection bias might still be present. Second, the low number of studies included in the review and meta-analysis could represent inadequate statistical power and bias due to the small sample size included in each comparison. In addition, most of the studies did not include a placebo intervention in addition to the standard treatment, which makes it difficult to determine whether the effects were driven by VR and not due to nonspecific effects. Third, there is great variability in the interventions and measurement procedures used among the studies. In addition, methodological concerns regarding the studies included, especially in terms of to performance and detection bias, should be considered when interpreting results. Finally, the studies included had short-term follow-up periods so, in the future, it would be necessary to carry out studies with long-term follow-up periods to conclude with security the extent of the benefits obtained with VR systems, and to understand how much time the benefits of this method last in comparison to the conventional treatment, since it could be possible that, in long-term follow-up periods studies, given the increase on the adherence that this method provides, a VR treatment could appear to be more effective than the conventional treatment. This has yet to be confirmed.

## 5. Conclusions

The results of the present review suggest that VR-based treatments are more effective than non-intervention in improving balance and fall risk in patients with MS, with a very low certainty of evidence. High level of bias and imprecision make it necessary to consider these findings carefully. In addition, VR also appears to be more effective than conventional rehabilitation, with a very low/low certainty of evidence. Future studies should evaluate the long-term effects of VR, as well as determine the best intervention parameters for its application in a clinical setting.

## Figures and Tables

**Table 1:** Characteristics for the extracted data.

Study	Participants	Groups and Treatments	Variables	Results
Brichetto et al., 2013 [39] (Italy)	START: (36). CG = 18/VRG = 18. END: (36). CG = 18/VRG = 18.	CG = They did static and dynamic exercises with 1 and 2 legs and with and without the balance board. VRG = They did balance exercises using the Wii Fit system with the “Wii Balance Board”.	Balance: BBS. Fatigue: MFIS. Postural valoration: Stabilometric platform.	There were significant differences between groups in favor of the VRG in the BBS (*p* < 0.05), in MFIS (*p* < 0.05), and in the stabilometric platform (*p* < 0.05).
Calabrò et al., 2017 [40] (Italy)	START: (40). RAGTG = 20/ RAGTG-VR = 20. END: (40). RAGTG = 20/ RAGTG-VR = 20.	RAGTG = They used the “Lokomat-Nanos” system. RAGTG-VR = They used the “Lokomat-Nanos” system + the “Lokomat-Pro” system, 5 times per week for 8 weeks.	Balance and mobility: BBS, TUG. Muscle spasticity: MAS. Ambulatory ability: EDSS. Stress response: COPE. Depression/symptoms somatization: HRDS. Physical and cognitive disability: FIM.	The RAGTG-VR showed better results in balance, mobility, and stress response, but there were no significant differences between groups BBS (*p* = 0.8) and TUG (*p* = 0.3).
Eftekharsadat et al., 2015 [41] (Iran)	START: (30). CG = 15/VRG = 15. END: (30). CG=15/VRG =15.	CG = They did not receive any treatment. VRG = They carried out a balance/postural control training program using the Biodex balance system, 2 times per week for 12 weeks.	Balance: BBS, TUG, Romberg Test, OSi. Muscle spasticity: MAS. Movement and muscle position: MMT. Risk of falls and postural stability: Biodex Balance System SD.	The VRG had significant improvements in TUG (*p* = 0.01) and in FRi (*p* = 0.002) and OSi (*p* = 0.04), but there were no significant differences between groups in BBS (*p* > 0.05).
Gutierrez et al., 2013 [42] (Spain)	START: (50). CG = 25/VRG = 25. END: (47). CG = 23/VRG = 24.	CG = Conventional physiotherapy, 2 times per week for 10 weeks for 40 min. VRG = They did 4 sessions of monitored telerehabilitation using Xbox 360^®^ for 10 weeks during 20 min each session.	Balance/postural stability and gait: SOT (CES, MCT, BBS, TBS). Fatigue perception: AVS. Vision: PREF, ViR. Somatosensorial level: VEST/SR.	There were significant differences in favor of the VRG in MCT (*p* = 0.003), in CES (*p* = 0.003), in BBS (*p* < 0.001), and in TBS (*p* < 0.001). Balance improved in both groups.
Hoang et al., 2015 [43] (Australia)	START: (50). CG = 22/SG = 28. END: (44). CG = 21/SG = 23.	CG = They continued with their usual physical activity. SG = They did at home-based exercises using the step and the open software “Stepmania” 2 times per week for 12 weeks for 30 min.	Risk of falls: CSRT, SST. Dynamic balance and mobility: TUG. Postural swinging. Gait capacity and speed: 10MWT, 6MWT. Cognitive capacity: TMT, SDMT. Divided attention: TUG + double tasking. Disability level: MSFC, 9-HPT.	The SG improved in the CSRT (*p* = 0.031) and in the SST (*p* = 0.011). The SG showed less postural swinging with open eyes (*p* = 0.023), + rapidity in 10MWT (*p* = 0.023) and a reduction in 9-HPT (*p* = 0.001).
Kalron et al., 2016 [44] (Israel)	START: (32). CG = 16/VRG = 16. END: (30). CG = 15/VRG = 15.	CG = Traditional physical training, 2 times per week for 6 weeks for 30 min. VRG = Same period y frequency but doing exercises using the “CAREN” system + the D-Flow software.	Static and dynamic balance: BBS, FSST. Stability: FRT. Fear of falls: FES-I. Swinging: “(CoP) Path Length”.	Both groups improved in the postural control (*p* = 0.024), FRT (*p* = 0.001), and FSST (*p* = 0.031). There were significant differences in favor of the VRG in FRT (*p* = 0.009) and FES-I (*p* = 0.021).
Khalil et al., 2018 [45] (Jordan)	START: (40). CG = 20/VRG = 20. END: (32). CG = 16/VRG = 16.	CG = At home balance exercises 3 times per week for 6 weeks. VRG = VR-based balance exercises 2 times per week for 6 weeks + the same as the CG.	Balance and mobility: BBS, TUG, 3-MWD. Gait speed: 10-MWT. Fatigue: MFIS. Quality of life: SF36; version 1. Balance perception and fear of falls: FESI.	The VRG showed better results compared to the CG in BBS (*p* = 0.012), MFIS (*p* = 0.008), and in several parameters of the SF36 (*p* < 0.05).
Lozano-Quilis et al., 2014 [9] (Spain)	START: (11). CG = 5/VRG = 6. END: (11). CG = 5/VRG = 6.	CG = Traditional physiotherapy treatment. VRG = They did balance and gait exercises using the Removi-EM system, 1 time per week for 10 weeks for 1 h.	Static balance: BBS, TBS, SLB. Dynamic balance: 10MWT, TUG. Treatment feedback: SEQ.	There were significant differences in favor of the VRG in TUG (*p* = 0.027), in BBS (*p* = 0.030), and in the SLB with the right foot (*p* = 0.033).
Nilsagård et al., 2012 [46] (Sweden)	START: (84). CG = 42/VRG = 42. END: (80). CG = 41/VRG = 39.	CG = They used the Nintendo Wii Fit Plus after the data of the investigation was recorded. VRG = They used the Nintendo Wii Fit Plus for 6–7.	Balance and mobility: TUG. Physical/psychosocial problems: MSIS-29. Double tasking capacity: TUGcognitive. Ability to take steps with height: FSST. Gait speed: 25TW./Gait capacity valoration: MSWS-12/Gait dynamic balance: DGI. Self-confidence: ABC. Strength and functional balance: TCS.	There were no statistically significant differences between groups. The individual results of the VRG were of a greater relevance compared to the results of the CG.
Ozkul et al., 2020 [47] (Turkey)	START: (51). CG = 17/BG = 17/ IVRG = 17. END: (39). CG = 13/BG = 13/ IVRG= 13.	CG = Jacobson’s progressive relaxation exercise 2 times per day, for 8 weeks. BG = Traditional balance training. IVRG = Balance training for 20 min using the “RAGU” system and the “Microsoft’s KinectV2”.	Balance: BBS. Postural control: “Biodex Balance System-BioSwayTM”. Mobility: TUG. Fatigue: FSS.	The IVRG and the BG improved the stability limits, postural control, mobility, and fatigue equally (*p* < 0.05). The CG did not improved the same way.
Peruzzi et al., 2016 [48] (Italy)	START: (25). CG = 11/VRG = 14. END: (25). CG = 11/VRG = 14.	CG = Treadmill-based training, 3 times per week for 6 weeks for 45 min. VRG = Treadmill + VR-based training using the WorldViz software, 3 times per week for 6 weeks for 45 min.	Balance and mobility: BBS, TUG. Gait capacity and speed: 6MWT, 10MWT. Disability: EDSS. Negotiation of obstacles: FSST.	The VRG showed significant improvements in BBS (*p* = 0.003) and FSST (*p* = 0.028), but without significant differences between groups.
Prosperini et al., 2013 [49] (Italy)	START: (36). GA = 18/GB = 18. END: (34). GA = 17/GB = 17.	GA = They did at home-based balance training using the “Nintendo Wii Balance Board” system every day for 12 weeks for 30 min. After, they had 12 weeks of no treatment. GB = The same, but the other way around.	Static balance: Strength platform/Dynamic balance: FSST. Disability evaluation: 25-FWT. Physical/psychological evaluation: MSIS-29.	The VR training proved to be effective regardless of the order of the treatment. The results for the static balance were positive (*p* = 0.016) and in the FSST as well (*p* = 0.034).
Robinson et al., 2015 [50] (UK)	START: (56). GC = 18/PTG = 18/ VRG = 20. END: (46). CG = 11/PTG = 15/ VRG = 20.	CG = They did not receive any treatment. PTG = Conventional physical training 2 times per week for 4 weeks for 40–60 min. VRG = Balance, aerobic, and muscle-strengthening exercises using the Wii Fit system during the same period of time and frequency as the PTG.	Postural swinging (Balance): Strength platform. Gait: “GAITRite™”. Technological acceptance: UTAUT questionnaire. Gait experience: FSS questionnaire. Gait own perception: MSWS-12 questionnaire. Perceived activity/disability: [WHODAS 2.0] questionnaire.	The VRG and the PTG improved in the standing postural swinging and in the WHODAS 2,0 questionnaire (*p* ≤ 0.001). There was a significant improvement in favor of the VRG in FSS (*p* ≤ 0.05). In the UTAUT, the VRG showed better results in comparison to the PTG, but without significant improvements. Gait did not improve significantly in any of the groups.
Russo et al., 2017 [51] (Italy)	START: (45). CG = 15/VRG = 30. END: (45). CG = 15/VRG = 30.	CG = Traditional physical training for 18 weeks. VRG = They used the “Lokomat-Pro” system 3 times per week for 6 weeks and later they did the same as the CG.	Balance and mobility: TBS, TUG. Disability: EDSS. Independence level: FIM. Depression: HRSD.	After 6 weeks, the VRG improved in all parameters: TBS and TUG (*p* < 0.001). The CG only improved in TUG. At the end of the treatment, the CG improved in all parameters, but the VRG only improved in TUG. In the reevaluation, the CG got worse in TBS and TUG and the VRG in TUG.
Thomas et al., 2017 [52] (UK)	START: (30). IG= 15/PG = 15. END: (28). IG = 13/PG = 15.	IG = They used the “Mii-vitaliSe” system for 12 months + the usual cares. PG = They used the same system but 6 months later, so their treatment lasted just 6 months.	Balance, gait, and mobility: 2MWT, Step Test, Steady Stance Test, i-TUG, Gait Stride-time Rhythmicity, Static Posturography. Physical activity: GLTEQ, ActivPAL3 tri-axial accelerometer. Quality of life: HADS, EQ-5D-5L, MSIS-29, FSI, SF-36 v.2. Individual capacity: SCI-ESES, MSSE. Manual dexterity: 9HPT.	The balance and gait tests showed a standardized size effect in favor of the intervention.
Yazgan et al., 2019 [53] (Turkey)	START: (47). CG = 15/BG = 16/ VRG = 16. END: (42). CG = 15/BG = 12/ VRG = 15.	CG = The participants were included in a waiting list to use the “Nintendo Wii Fit” once the study was finished. BG = Balance exercises on the Balance Trainer. VRG = Balance exercises using the “Nintendo Wii Fit”.	Balance: BBS. Mobility: TUG. Fatigue: FSS, 6MWT. Quality of life: MusiQol.	The VRG showed better results compared to the CG in BBS (*p* < 0.001), TUG (*p* = 0.005), and 6MWT (*p* = 0.008). The BG showed better results than the CG, but not as good as the results of the VRG. The VRG showed better results than the BG in BBS (*p* = 0.038).

**Table 2:** PEDro scores for each study.

Items												
	1	2	3	4	5	6	7	8	9	10	11	Total
Gutierrez et al. [42]	1	0	0	1	0	0	1	1	0	1	1	5/10
Peruzzi et al. [48]	1	1	0	1	0	0	1	0	0	1	1	5/10
Nilsagård et al. [46]	1	1	1	1	0	0	1	1	0	1	1	7/10
Eftekharsadat et al. [41]	1	1	0	1	0	0	1	1	0	1	1	6/10
Thomas et al. [52]	1	1	1	1	0	0	0	1	1	1	1	7/10
Calabró el al. [48]	1	1	1	1	0	0	1	1	1	1	1	8/10
Khalil et al. [45]	1	1	1	1	0	0	1	0	0	1	1	6/10
Kalron et al. [44]	1	1	1	1	0	0	1	1	0	1	1	7/10
Russo et al. [51]	1	1	1	1	0	0	1	1	0	1	1	7/10
Yazgan et al. [53]	1	1	0	1	0	0	0	1	0	1	1	5/10
Ozkul et al. [47]	1	1	0	1	0	0	1	0	0	1	1	5/10
Lozano-Quilis et al. [9]	1	1	0	1	0	0	0	1	0	1	1	5/10
Hoang et al. [43]	1	1	1	1	0	0	1	1	1	1	0	7/10
Prosperini et al. [49]	1	1	1	1	0	0	1	1	0	1	1	7/10
Brichetto et al. [39]	1	1	0	1	0	0	1	0	0	1	1	5/10
Robinson et al. [50]	1	1	0	1	0	0	0	0	1	1	1	5/10

**Table 3:** Certainty of evidence.

Certainty Assessment						No. of Participants		Effect		Certainty
Outcome (No. of Studies)	Study Design	Risk of Bias	Inconsistency	Indirectness	Imprecision	Interv.	Control	Relative (95% CI)	Absolute (95% CI)	
Balance (BBS)										
*Vs conventional*	RCT	Serious	Not serious	Not serious	Serious	126	121	-	0.63 (0.34–0.92)	Low
*Vs no intervention*	RCT	Serious	Serious	Not serious	Serious	59	58	-	0.94 (0.21–1.87)	Very Low
Risk of falls (TUG)										
*Vs conventional*	RCT	Serious	Serious	Not serious	Serious	114	95	-	−0.55 (−1.07–0.04)	Very Low
*Vs no intervention*	RCT	Serious	Serious	Not serious	Serious	121	120	-	−0.87 (−1.52–−0.23)	Very Low

## Data Availability

Not applicable.

## Supplementary Materials

The following supporting information can be downloaded at: https://www.mdpi.com/article/10.3390/ijerph192114192/s1, Supplementary Materials S1, Search equations; Supplementary Materials S2, Synthesis funnel and Doi plot (LFK index) for BBS variable (VR vs. conventional treatment) to assess the presence of publication bias; Supplementary Materials S3, Synthesis funnel and Doi plot (LFK index) for BBS variable (VR vs. no-treatment) to assess the presence of publication bias; Supplementary Materials S4, Synthesis funnel and Doi plot (LFK index) for TUG variable (VR vs. conventional treatment) to assess the presence of publication bias; Supplementary Materials S5, Synthesis funnel and Doi plot (LFK index) for TUG variable (VR vs. no-treatment) to assess the presence of publication bias.
