# Supplementary material for: Effectiveness of Virtual Reality on Balance and Risk of Falls in People with Multiple Sclerosis: A Systematic Review and Meta-Analysis
Source: Int J Environ Res Public Health. 2022 Oct 30;19(21):14192. doi: 10.3390/ijerph192114192 (PMC9656689; doi:10.3390/ijerph192114192)

Supplementary Data

Supplementary Material S1. Search equations.

("Virtual Reality"[MeSH Terms] OR "Immersive virtual reality"[All fields] OR “Wii Balance Board,"[All fields] OR “Exergaming”[All fields] OR “Nintendo Wii™,” [All fields] OR “Augmented Reality” [All fields] OR “Video Games”[All Fields]) AND ("Multiple Sclerosis"[MeSH Terms] AND ("Balance"[MeSH Terms] OR "Postural Balance"[MeSH Terms] OR "Gait"[MeSH Terms] OR “Walking Speed”[MeSH Terms] OR “Risk of falls”[All Fields] OR “Motor rehabilitation”[All Fields] OR “Neurorehabilitation”[All Fields] OR “Functional recovery”[All Fields] OR “Sway Area”[All Fields])

Supplementary Material S2. Synthesis funnel and Doi plot (LFK index) for BBS variable (RV vs conventional treatment) to assess the presence of publication bias.

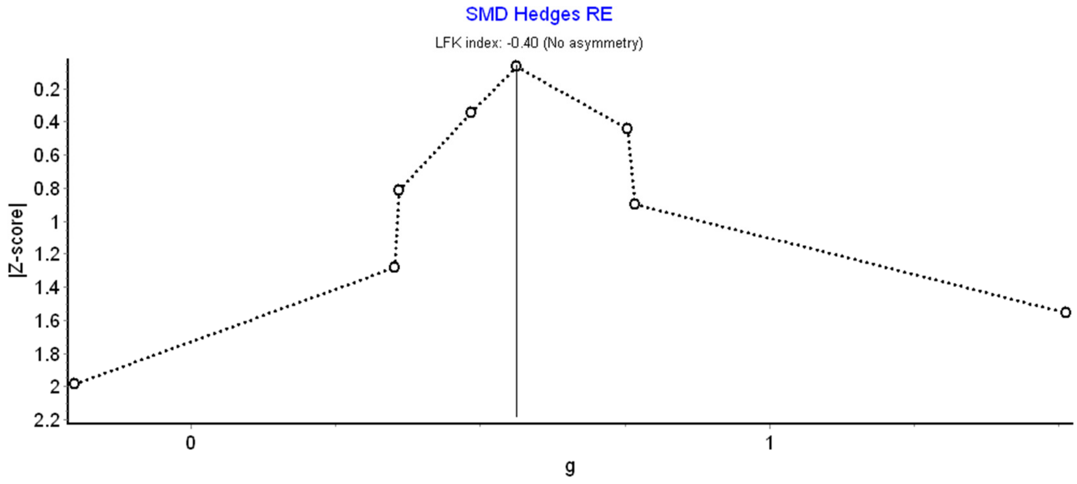

Supplementary Material S3. Synthesis funnel and Doi plot (LFK index) for BBS variable (RV vs no-treatment) to assess the presence of publication bias.

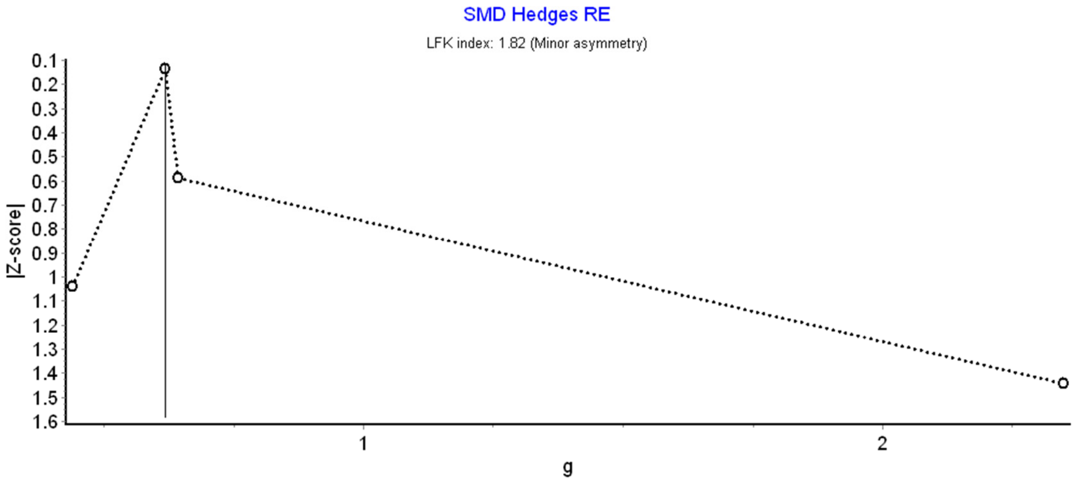

Supplementary Material S4. Synthesis funnel and Doi plot (LFK index) for TUG variable (RV vs conventional treatment) to assess the presence of publication bias.

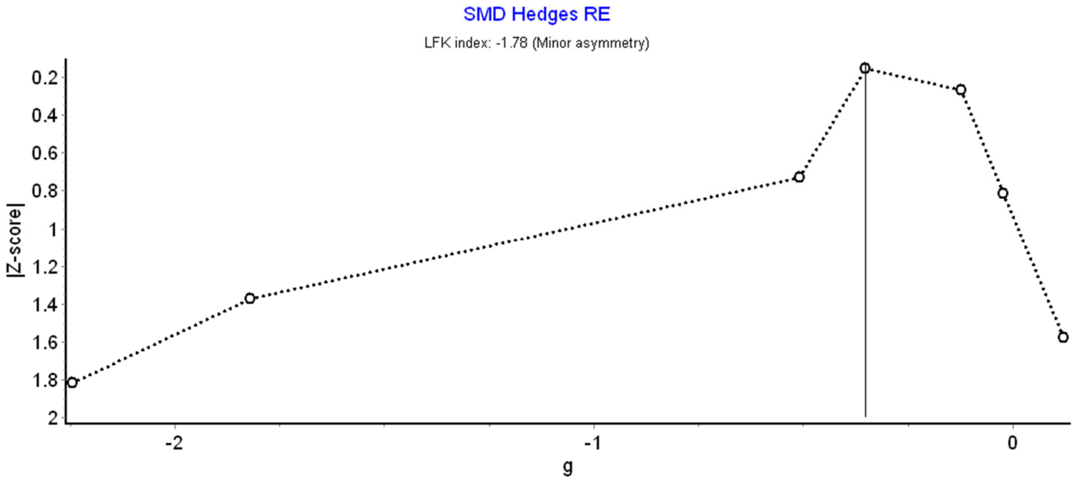

Supplementary Material S5. Synthesis funnel and Doi plot (LFK index) for TUG variable (RV vs no-treatment) to assess the presence of publication bias.

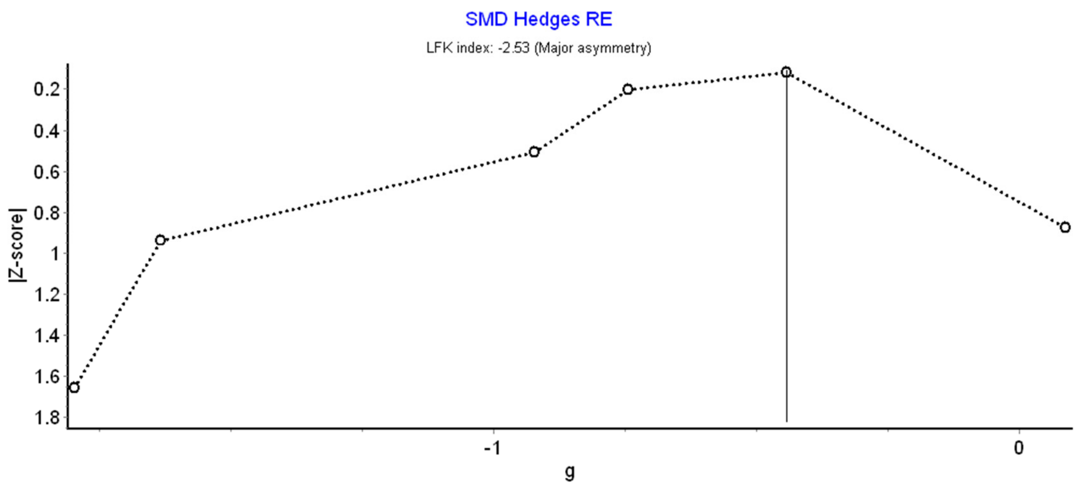

Supplement: Supplementary file 1 [file ijerph-19-14192-s001.zip › ijerph-1958946-supplementary.pdf]
